# Neuroimmune Mechanisms in Signaling of Pain During Acute Kidney Injury (AKI)

**DOI:** 10.3389/fmed.2020.00424

**Published:** 2020-08-07

**Authors:** Aprajita Gupta, Dev Kumar, Sanjeev Puri, Veena Puri

**Affiliations:** ^1^Centre for Systems Biology & Bioinformatics, Panjab University, Chandigarh, India; ^2^Department of Biochemistry, Postgraduate Institute of Medical Education and Research, Chandigarh, India; ^3^Department of Biotechnology, University Institute of Engineering and Technology, Panjab University, Chandigarh, India

**Keywords:** acute kidney injury, neuroinflammation, pain, neuropeptides, CGRP, TNF-α, inflammation

## Abstract

Acute kidney injury (AKI) is a significant global health concern. The primary causes of AKI include ischemia, sepsis and nephrotoxicity. The unraveled interface between nervous system and immune response with specific focus on pain pathways is generating a huge interest in reference to AKI. The nervous system though static executes functions by nerve fibers throughout the body. Neuronal peptides released by nerves effect the immune response to mediate the hemodynamic system critical to the functioning of kidney. Pain is the outcome of cellular cross talk between nervous and immune systems. The widespread release of neuropeptides, neurotransmitters and immune cells contribute to bidirectional neuroimmune cross talks for pain manifestation. Recently, we have reported pain pathway genes that may pave the way to better understand such processes during AKI. An auxiliary understanding of the functions and communications in these systems will lead to novel approaches in pain management and treatment through the pathological state, specifically during acute kidney injury.

## Introduction

The kidney is a complex organ, packing millions of nephrons and cells with specialized functions that are essential for homeostatic balance of the body. A number of factors that lead to origin and progression of kidney injury includes environmental factors, toxins, other organ pathological cues as well as the genetic factors. The cellular crosstalk by these molecules together with different cellular mechanisms that afflict AKI and thereafter its neurological impairments such as nociception are incompletely understood. In this quest, we have recently reported the putative genes that participate in neuroimmune interactions during pain perception of AKI (1). Further enriching our knowledge of neuro-immune interaction in renal injury could lead to new therapies for inflammation induced pain and other neurological impairments.

Acute kidney injury (AKI) was first discovered during world war- II, wherein the war victims with crush injury led to the acute impairment of renal function (2). AKI accounted for the most common reason for consultation in nephrology. The epidemiology of AKI is variant with global economic scenario in developing and developed countries (3). Using the KDIGO definition, 1 in 5 adults (21.6%) and 1 in 3 children (33.7%) experience AKI worldwide. The pooled AKI-associated mortality rate is 23.9% in adults and 13.8% in children (4). It has been estimated that every year 2 million people die of AKI (5).

AKI is a syndrome resulting in low glomerulus filtration rate (GFR) and accumulation of nitrogenous wastes leading to impairment in hemodynamic regulations. Biochemically, AKI is characterized by increase in blood urea nitrogen (BUN) and creatinine levels (serum creatinine ≥0.3 mg/dl or a 1.5-fold increase in serum creatinine levels within 48 h) (6).

Etiologically, AKI is clinically divided into three classes: pre-renal, intra-renal/intrinsic and post-renal. Pre-renal AKI is a condition due to external factors like hypotension, toxins, renal vascular thrombosis, cardiac failure, hypocalcaemia, activation of neurohumoral axis and increase in renal vascular resistance leading to an impairment in functioning of the kidney (7). Intrinsic kidney injury is a condition that occurs when the damage occurs due to loss of function in glomeruli, renal tubules or interstitium. Post-renal condition occurs due to an obstruction in the urinary tract for example due to kidney stones, blood clots in the ureters or urethra, or due to cancer of the prostate, cervix, or colon (8).

Patients of AKI usually report with symptoms like pain in the flanks (9), nausea, little urine leaving the body, swelling in legs, ankles and around the eyes (10). They have a high risk of acquiring chronic kidney disease, likelihood of end stage renal disease, symptoms of encephalopathy, cognitive impairments and increased risk of mortality (11). Uremic neuropathy is a disorder observed in the AKI patients as a later stage complication, leading to sensory loss in lower limbs, motor impairments and pain (12–14).

The intrinsic pathophysiology behind all these complications of AKI is complex. It is consequential to interrupted flow into renal tissues, toxins, or obstruction to the urine flow or presence and accumulation of toxins that activate the immune system leading to inflammation, reduction in the blood flow in cortex and medulla along with disruption of tubular kidney cells thereby contributing to low GFR and low urine output (15). This activation can lead to virtually all components of innate and acquired immune systems causing dysfunction of both tubular and endothelial cells. In our previous work, we have evidenced an increase in the gene expression for TNF-α, relA, p53 and NFκβ upto 12 h in folic acid induced AKI along with biochemical changes in kidney (16). In concordance with other reports, we have also observed inflammation in glomeruli, tubular dilations with flattened epithelium, swelling in primary and secondary pedicels, significant deprivation of erythrocytes (RBCs) and blebbing in villi (17).

The co-existence of a number of receptors in nervous and immune system advocates for functional amalgamation of these two discrete systems. The immune system triggers the release of mediators that can modulate the neuronal activities directly or can influence through specific receptors, expressing effects from peripheral nerve modulation to alteration in complex cognitive system through the hormonal and neuronal pathways. The nociceptors also release neuropeptides and neurotransmitters which consequently generate the action of more immune responses. Such neuroimmune interactions play a key role in the initiation as well as propagation of peripheral inflammation (18). A well-known example of the neuroimmune association with inflammation is the hypothalamic-pituitary-adrenal axis which may be upregulated in kidney disease (19).

AKI patients are by and large reported to have cognitive problems. It has been shown that elderly patients who have impairment in kidney functions tend to have more rapid rate of cognitive decline (20). The soluble inflammatory proteins formed during kidney injury lead to hippocampal inflammation and uremic encephalopathy, respectively (21). Nociception is influenced by cytokines like TNF-α, IL-6, and IL-10 which are abundantly released during the kidney injuries. This elicits the COX-2 to release PGE2 in the endothelial cells in CNS (22). Thus, the cognitive impairment and cerebrovascular disturbance in AKI can be due to haemodynamic instability, changes in body fluids, and/or cerebral oedema (20).

Pain serves some obvious physiological functions for the body, such as warning of potentially dangerous stimuli or drawing attention to injured tissue (23). The message passed from these molecules serves as a trigger for the activation of immune response and recruitment of defense and repair material vital for the tissue healing. However, the lingered pain persisting even after disappearance of stimulus is a pathological response. During AKI, the intensity of pain varies from hyperalgesia (mild pain), to chronic pain depending on the extent of AKI. Celsus defined pain as one of the cardinal signs of inflammation (24). Certain studies have shown that neuro-immune interactions influence and contribute to phenotypic manifestations of pain, mediated by nociceptor neurons (25).

**Figure 1:**
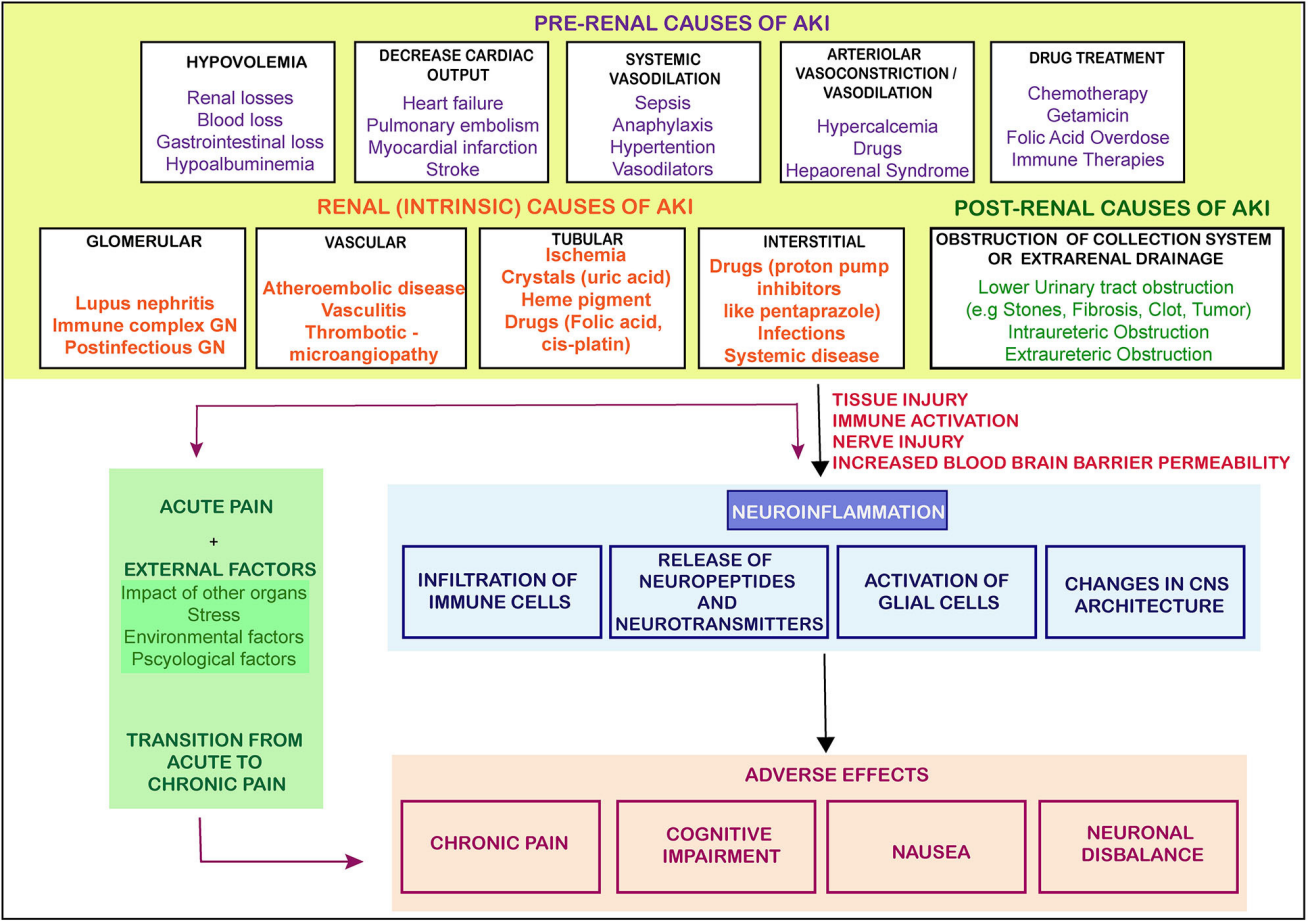
The causes and flow of pain signals during AKI. The figure shows the causes of AKI and the signals from the injured kidney leading to neuroinflammation and other adversities. There are three main categories of AKI which lead of progression of AKI toward pain, CKD or other adverse effects. GN- glomerular Nephritis (The arrows represent the induction of a process).

This bidirectional relationship is associated with multiple outcomes contributing to peripheral nociceptive sensitization by releasing soluble factors and paving an interaction directly with nociceptors. The nociceptors are ideally positioned as the first responders of pathogens and tissue injury, expressing their response by inducing pain and other behavioral changes in the body, leading to avoidance of noxious stimuli. Thus, influence of the nervous system on the immune system and vice versa is complex, multidirectional, and may be associated with multiple outcomes. We have suggested a model representation of all such possibilities in Figure 1 suggesting nociception factors in AKI.

Currently there is no specific treatment for this devastating clinical syndrome, which reflects in part, the complexity of causes and relatively poor understanding of the pathophysiology of this disease. In this review we are highlighting the entwinement and intricate interplay between the immune system and the nervous system to explore the putative messengers and the pathways which lead to establishment of pain following inflammation due to AKI. Together this approach can be used as possible pharmacological platform to manage the pain during AKI.

## Immune System Mediated Regulation of Pain During AKI

The immune system contributes to the initiation of renal disease. Renal diseases which are associated with dysfunction of immune homeostasis can be categorized according to direct or indirect immune-mediated kidney injury. Direct immune-mediated renal disease comprises the immune system aiming at specific kind of antigens within the kidney, while in indirect immune-mediated renal disease the kidneys become victim of infiltrating cells resulting from dysregulatory immune system regulation. There is significant intersection of the mechanisms by which both direct and indirect immune-mediated systems aid in pathways of kidney diseases (26).

During AKI an external stimulus activates intra renal immune cells. Damaged renal epithelial cells or necrotic cells, or part of the kidney with lowest oxygen tension activate their stress response pathways, leading to secretion of cytokines, inflammation and vasoactive factors (27). The necrotic and renal epithelial cells (mostly in outer medulla) release DAMPs activating the resident macrophages and DCs. This further activates immune cells and leads to infiltration of leucocytes leading to a triggered immune response. In a moderate injury, this activation of immune cells leads to repair and restoration of homeostasis. In alternate process, the aggravated activation of immune response contributes further to the development and progression of CKD, kidney fibrosis, and/or ESRD. This activation may even lead to transmission of inflammation to distant organs (26).

**Figure 2:**
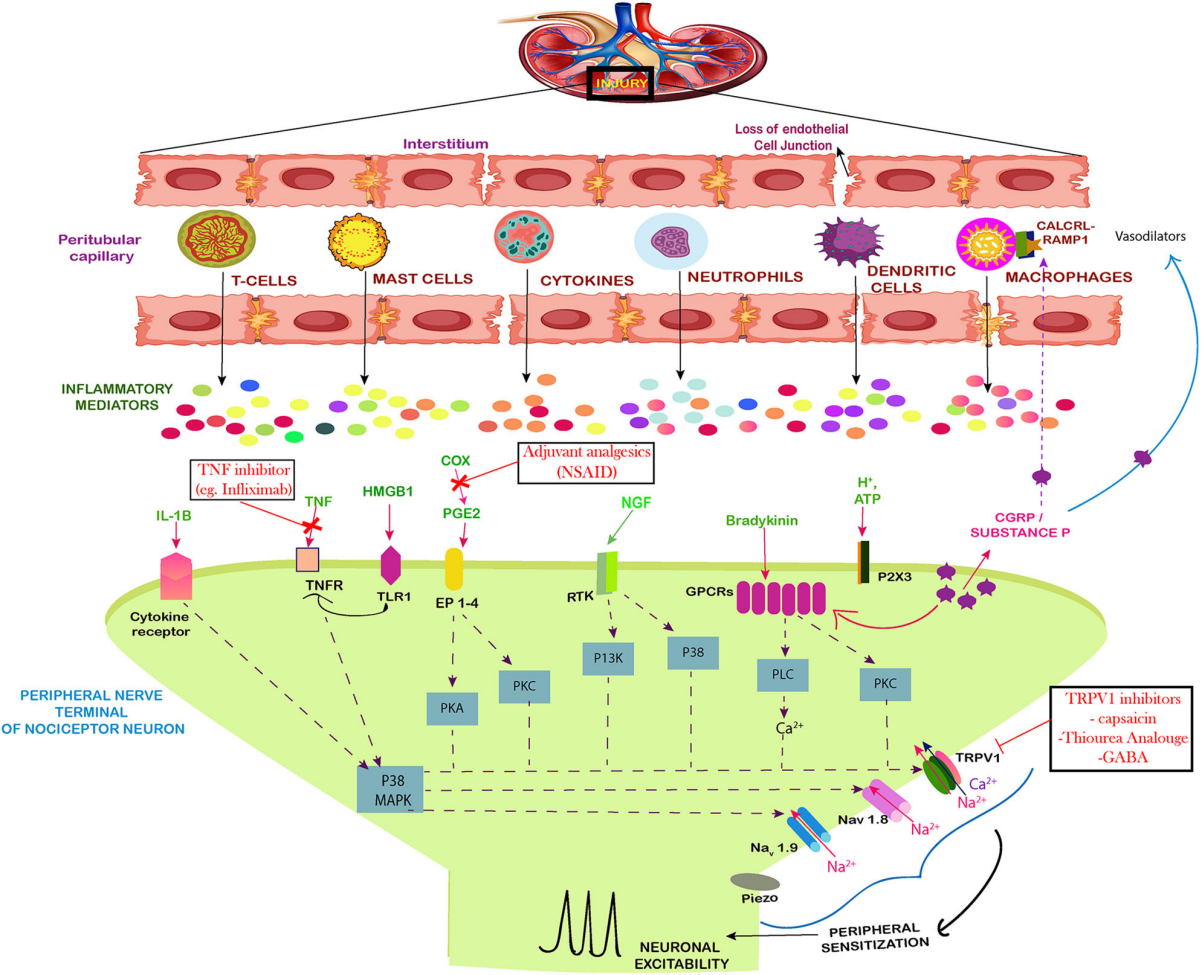
Neuro-immune interaction contributing to the establishment of pain during AKI. The figure shows the injured kidney secreting the immune cells and mediators that induce peripheral sensitization of nociceptor sensory neurons leading to pain. During inflammation induced by the injury, there is a loss of the cell architecture of the kidney tissues. This leads to release of specific immune cells, signal mediators, growth factors and inflammatory mediators. Together the influx of these cells leads to phosphorylation and/or gating of sodium ion channels Na_v_1.7, Na_v_1.8, Na_v_1.9, as well as the cation channels TRPV1, summing up to increased peripheral sensitization by action potential generation via calcium signaling. The Prostaglandin E_2_ (PGE_2_) also act via their receptors (EP1–4) to aid in peripheral sensitization by similar path. The tumor necrosis factor alpha (TNF-α) and IL-1β produced by mast cells, macrophages, and neutrophils also sensitize nociceptors mainly via calcium signaling. The same route is followed by NGF and GPCR signaling which act on the calcium channels. Along with these, the High mobility group box 1 protein (HMGB1) also activates the toll like receptors and increases expression of TRPV1 which mediates the pain signaling. The drugs used against pain in AKI have also been included.

The activated immune system has the strong drive in pain establishment and regulation by releasing molecular mediators that can stimulate the neuronal system (Table 1). The AKI stimulated immune system, activates the receptors and ion channel of nerve terminals and neurons (45). Nociceptor neurons express receptors for immune-cell, mast cells, antibodies, cytokines, and growth factors. Upon the activation of these receptors, the cell bodies within the dorsal root ganglion (DRG) relay signals to the CNS to be processed as pain. This is achieved by mediating signals via ion channels such as TRPV1, sodium channels, cytokine receptors and GPCRs etc. (Figure 2). This results in the peripheral damage and hyper activity of the primary sensory neurons leading to neuroinflammation. Neuroinflammation occurs in both PNS (peripheral nervous system) and CNS (central nervous system) and is characterized by the infiltration of macrophages, neutrophils and cytokines. The peripheral neuroinflammation brings about the changes in the activity of the neuropeptides, neuro transmitters and neuronal functions but does not cause significant neuronal loss in the CNS (46). In acute inflammation the pain progresses with the inflammation and demolishes as the inflammation moves toward recovery.

**Table 1:** Peculiar roles of immune components in AKI.

TNF-α	(16)	Important mediator of inflammatory tissue damage.
	(28)	Higher expression in brain during injury.
	(29)	Reticulates to interact with other organs and mediate neuroinflammation.
IL-1β	(30)	Early biomarker of AKI.
	(31)	Contributes in inflammation, recruitment of neutrophils and activation of Leucocytes.
	(32)	IL-1β and TNF-α together sensitize nociceptors by reducing the action potential.
Chemokines	(33)	Attract neutrophils, T lymphocytes and induce apoptosis.
	(34)	Involve in neuron–microglia interactions to induce pain signals.
		Modulate the release of neurotransmitters in brain.
T-cells		
CD^4+^	(35)	Increase in the initial hours of injury and decreases in the later hours.
	(36)	Involved in the splenic cholinergic anti-inflammatory pathway to mediate the reno-
		protective effect of ultrasound pre-treatment in renal IRI.
CD^8+^	(37)	Traffic into ischemic and injured kidneys.
		Produce more IFN-γ in injured kidneys when compared with normal kidneys.
T_reg_	(38)	Enhance repair process in later stage of injury.
	(39)	Decrease macrophage infiltration and aid in renoprotection in AKI.
NKT cells	(40)	Mediates injury by secreting several cytokines such as IL-4, IL-10 and IFN-γ.
	(41)	Paradoxically, some reports have evidenced reno-protection in AKI.
Neutrophils	(42)	Infiltrate into injured kidneys and generate reactive oxygen and nitrogen species.
	(43)	The process of infiltration is aided by neutrophil elastase, tissue type plasminogen activator, hepatocyte growth factor and CD44.
Macrophages	(44)	Infiltrate in outer medulla.
		Contribute to initial injury and fibrosis.
		Perfectly contribute in renal function impairment.
NK cells	(41)	Contribute to renal Injury by hitting tubular epithelial cells.
		Promote and maintain inflammation during AKI.

*The pivotal immune system components TNF-α (16), (28), (29), IL-1β (30), (31), (32), chemokines (33), (34), CD^4+^ (35), (36), CD^8+^ (37), T_reg_ (38), (39), NKT (40), (41), Neutrophils, (42), (43), Macrophages (44), NK cells (41) have been pointed out in the table. AKI, acute kidney injury; IFN, interferon; NK, natural killer; IL, interleukin; IRI, ischaemia–reperfusion injury; TNF, tumor necrosis factor; T_reg_ cells, regulatory T cells*.

### Role of Cytokines in Pain Regulation During AKI

Kidney is inhabited by a lower concentration of resident immune cells under normal circumstances but after the injury multiple immune cells and molecules are recruited by indirect or direct immune mediated pathways, which contribute inflammatory microenvironment. The injured kidney tends to infiltrate these cells, cytokines and intracellular proteins such as High mobility group protein B1 (HMGB1) to cause neuronal excitability (Figure 2). Studies have demonstrated that tubular epithelial cells (TECs) are extremely susceptible to intrinsic oxidative stress and play a pro-inflammatory role in kidney injury (47). The tubular epithelia produce TNF-α, IL-1, IL-16, IL-8, TGF-β, MCP-1, RANTES, ROS, and fractalkines (48). TNF-α is also produced by macrophages, lymphocytes, endothelial cells, epithelial cells, Schwann cells, mesangial cells, and podocytes in kidney (49). TNF-α is capable of inducing apoptosis and inflammation in renal epithelial cell (16) and can stimulate the production of reactive oxygen species (50). TNF-α mediates its function through TNFR1 and induces neuronal excitability in dorsal root ganglia (DRG) through p38 signaling (Figure 2). In response to glutamate, TNF-α enhances neuro-excitability and rapidly up-regulates the membrane expression and conductance of α-amino-3-hydroxy-5-methyl-4-isoxazolepropionic acid receptor (AMPA) receptors in neurons (51). TNF-α significantly upregulates the kidney type glutaminase (KGA) in cytosol and extracellular fluid of human neurons, exposing glutaminase as a potential component of neurotoxicity during inflammation (28). Along with this, the levels of Granulocyte–colony stimulating factor (G-CSF), glial fibrillary acidic protein (GFAP), microglial cells and Keratinocyte cells (KC) increases in the brain in response to AKI (52) due to the recruitment of neutrophils at the site of neuronal damage. The protein level of TNF-α has also been estimated to increase in the brain during acute kidney injury. In CNS, TNF-α is released by microglia and astrocytes which induces pain. TNF-α initiates, brain derived neurotrophic factor, BDNF-trkB feed-forward pathway in peripheral sensory neurons, and this up-regulation of BDNF-trkB system may participate in pain sensitization (53). Hence, TNF-α which is associated with increased inflammation during AKI is also considered as a bridge between immune response and neurons.

IL-1β was one of the first cytokines, whose role was reported both in inflammatory neuropathic pain and impairment of the signaling pathways of nerve injury (30). It is expressed by microglia and astrocytes in the spinal cord in low concentrations under the normal physiological conditions. However, just like other inflammatory molecules, their ratio increases on induction of an injury. IL-1β contributes in the inflammation and causes the mobilization of neutrophils from bone marrow, initiating response of proteins such as C-reactive protein, by activating endothelial cells and by activation of all classes of leucocytes. The expression of IL-1β is increased in the injured sciatic nerve, DRG, and spinal cord produced in microglia in the CNS (54) and appeared to be a contributing factor to the pain state (55).

Both TNF-α and IL-1β serve to activate or sensitize nociceptors by reducing the action potential. IL-1β induces hyperalgesia by sensitizing the nociceptor neurons through phosphorylation of Na_v_ 1.8 sodium channel by p38 MAPK signaling. TNF-α also induces neuronal sensitization by phosphorylation of Na_v_ 1.8 and Na_v_1.9 through same signaling (32). Apart from generating this inflammatory pain state, the signaling of neuronal TNF-α and IL-1β leads to production of chemokines that are linked to the peripheral nerve injury (56). IL-1β modulate neuronal excitability by causing an increase in TRPV1 expression sodium channels, GABA receptors, and NMDA receptors (57). Other interleukins associated with AKI are IL-34 (58), IL-5 which sensitizes nociceptor neurons by IL-5 receptors and IL-1 which induces transcription of cyclooxygenase-2 (COX-2) and further leads to the production of prostaglandin E2 (PGE2). The proinflammatory cytokines IL-6 and IL-8 are found elevated in serum of patients at early stage of AKI. Further IL-6 upregulates endothelial CXCL1 production and aids in mediation of lung inflammation and injury after AKI (59). IL-17A is observed to promote inflammation through induction of chemokine expression and induces rapid phosphorylation of Erk and protein kinase B in DRG (60).

### Role of Chemokines in Pain Regulation During AKI

The role of chemokines has been found to be potentially important for the development and maintenance of painful neuropathies. Chemokines are synthesized in response to kidney injury and also by nociceptive neurons. The chemokines are induced by cytokines, complement activation, ROS or NF-kB signaling like pathways. The animal model and patients' studies have revealed the role of chemokines and chemokine receptors in inflammatory kidney diseases. They aid in the development and maturation of leukocytes, metastasis, wound healing and angiogenesis. Chemokine-mediated neuron-glial interactions is bidirectional; while the neurons express chemokines, the glial cells express the receptors for them and vice-versa. Their receptors are well-oriented to mediate and promote the signaling interactions between neurons and glial cells (61).

The production of pro-inflammatory chemokines, macrophages inflammatory factor-2 (MIP-2) (also called CXCL2) and keratinocyte-derived chemokine (KC) (also known as CXCL1) are neutrophil chemoattractant which attract both neutrophils and T lymphocytes to the site of inflammation. They are known to mediate AKI (42) and also attract neutrophils and macrophages to the injured kidney (Figure 2). CXCL1 has been reported to promote neutrophil recruitment and induction of apoptosis (62). CXCL1 and CXCR2 together mediate astrocyte–neuron interactions and play an important role in the maintenance of neuropathic pain (63). The chemokine CX3CL1 and CX3CR1 are involved in neuron–microglia interactions in the spinal cord to promote chronic pain (64). Along with these, chemokines such as CCL2, CCR2, and CCL7 which are produced during AKI, facilitate neuropathic pain by inducing spinal cord astrocytes (65). The interaction between CXCL16 and CXCR6, recruit natural killer cells (NK) to the site of injury and promote inflammation. CXCL16 is also known to modulate the release of neurotransmitters in brain (33). The chemokines RANTES, SDF1α, MCP1, and fractalkine can produce excitation and can act on a group of related G-protein-coupled receptors (GPCRs), which further interact with pain pathways (66).

### Role of Immune Cells in Pain Regulation During AKI

Neutrophils (or polymorphonuclear leukocytes) are an essential part of the innate immune system and their migration is associated with inflammatory pain. They are the most abundant and the first set of immune cells at the site of injury. Their cytoplasm comprises of granules and secretory vesicles that can release a varied range of defense molecules including cytokines, ROS, bactericidal proteins and chymotrypsin-like serine proteases (NSPs) in their granules. The first step in their action is the migration toward an inflammatory point by gradient of chemo-attractants and adhesion to the surface of the vascular endothelium to accumulate at the site of injury (67). This recruitment is influenced by the nerve terminals and it is termed as neurogenic inflammation. The primary afferent neurons generate an impulse which spreads through nerve terminals to release VIP, Substance P and CGRP like neuropeptides. A line of evidence shows that NGF triggers CGRP expression in nociceptive transmission and pain through the pathways regulated by Erk and CREB (68) (Figure 2). CGRP shows a decrease in the recruitment of neutrophils by inhibiting chemokine production (69) and on the contrary CGRP has been observed to promote neutrophil adherence to endothelium (70). Neutrophils release mediators such as defensins and cytokines in order to contribute to inflammatory hyperalgesia. In normal scenarios, the neutrophils are not found in the nerves but during a pathological abnormality they have been found at the site of injury in peripheral nerves.

Macrophages and monocytes are activated and increased in number during AKI. During initial stage of kidney injury, macrophages M1 aid in the promoting tubular injury by recruiting cytokines such as IL-1β, TNF-α, IL-12, IL-18, and IL-23 (71). The macrophage M2 is generated from polarization of macrophage. It has been reported that M2 macrophages curtail inflammation, support structural regeneration, functional recovery and aid in the recovery phase by tubular proliferation (72). The neuropeptide CGRP relays anti-inflammatory effects on myeloid cells leading to decrease in cytokine formation, decrease in oxidative stress, decrease in lipid peroxidation of membrane and upregulation of IL-10, the anti-inflammatory cytokine in macrophage and dendritic cells (73). In myeloid cells (Figure 2) this complex signals the cAMP-PKA dependent upregulation of IL-10. It is interesting to see that CGRP as a pain mediator, plays a role contrast to its nature, in negative feedback control of macrophage-nociceptor interaction and reduce further the nociceptor sensitization to reduce the pain stimuli.

Mast cells are involved in AKI and fibrosis and relocate themselves to injured area in response to inflammatory cues. Mast cell-derived TNF is known to sensitize meningeal nociceptors and thereby induce neuroinflammation (74). They are strong associates of the neuroimmune correspondence as they are capable of migrating across an intact blood–brain barrier (BBB). Mast cells express receptors for CGRP and the released CGRP can stimulate degranulation and activation of mast cells (75).

T-cells are important early mediators of kidney injury which have a significant role in inflammatory responses mediated by neutrophils. T-cells may become activated in kidney injury by inflammatory cytokines, reactive oxygen species or by specific antigens. The T-helper cells (Th) CD3+ and CD4+ recognize peptide antigens and promote the activity of other inflammatory cells (35). Several studies in experimental AKI have suggested that cellular therapy with DCs and regulatory T cells could promote tissue repair.

### Role of Complement System in Pain Regulation During AKI

The complement system also has a role in pain and hyperalgesia mediation (76). The deposition of C3b and correlation of complement activation with pain was first observed in 1975 in kidney patients (76). The activation of complement system results in the production of the anaphylatoxins C3a and C5a, both of these are directly involved in neuropathic pain and sensitization of C fiber nociceptor, by C5a binding to its receptor C5aR. This further trigger the release of cytokines that attract macrophages and neutrophils at the site of kidney injury. The role of alternative complement pathway in acute tubular injury has lately been discovered in sheep model, after a high dose of anti-inflammatory drugs (77).

### Role of Lipid Mediators in Pain Regulation During AKI

Prostaglandins (PGs) are abundantly produced in the kidneys and contribute to the kidney diseases by playing an important role in inducing the inflammatory response. COX-1 is significantly expressed in the glomerulus, collecting duct, medullary interstitial cells and mesangial cells. The macula densa region of kidney along with glomerulus, mesangial cells, medullary interstitial cells and thick ascending limb predominantly express COX-2 which plays a significant role in kidneys by regulating renin release and metabolism of salt and water (78). The variations appearing from different experimental conditions and models exhibit dual roles of COX in AKI. While some reports have evidenced the benefits of COX-2 inhibitors, other reports show that during AKI, the imbalance of fluid secretion and intra-renal hemodynamic inhibits COX-2, accounting for impairment in the renal function (79).

Arachidonic acid released from the phospholipids is converted to PGI_2_, PGE_2_, PGF_2_ and thromboxane A_2_, in the kidneys by cyclooxygenase enzyme. PGE2 and PGF2 are predominantly produced in the renal medulla. The prostaglandin PGE2 is the most abundant metabolite of the renal arachidonic acid. The microsomal PGE synthase 1 (mPGES-1), is considered to play a major role in chronic and acute kidney injuries as well as the in the mediation of inflammatory response (80). The activation of TNF-receptors induces prostaglandin synthesis leading to activation of nociceptive neurons and thereby stimulate pain (81). The enhanced endothelial release of PGI_2_ reduces acute renal injury by inhibiting TNF-α production and leukocyte activation (82). PGE2 functions via activating its four types of receptors (EP1, EP2, EP3, EP4). Amongst these, the EP4 receptor has been the best identified subtype and most abundant in almost all types of renal cells (Figure 2). The presence of EP4 in the distal convoluted tubule and the cortical collecting duct increases the trafficking of AQP2 and increase the water reabsorption possibly via both cAMP/PKA and Erk pathway (83). This points PGI2 as an important artifact of COXs, which couples with its receptors in signaling of AKI. Prostacyclin (PGI2) is also one of the main products of COXs pathways and has a potent role in the renal hemodynamic, rennin release and tubular transport. Its defect could be mediated by oxidative stress as generated during AKI and further aid in the progression toward CKD and later on to end-stage renal disease (ESRD) (84).

## Nervous System Mediated Regulation of Pain During AKI

### Reticulation of Nerves in Kidney

**Figure 3:**
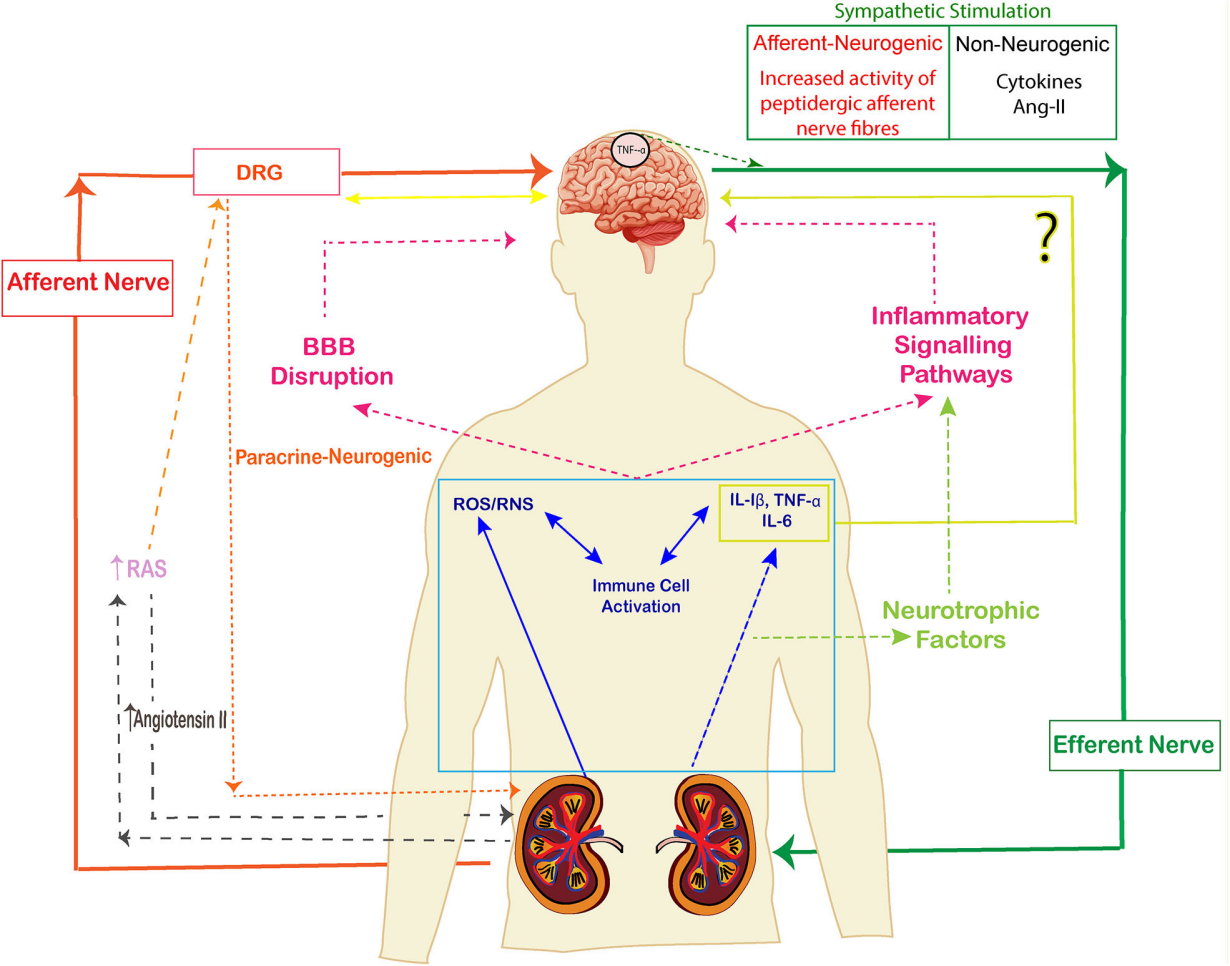
The signaling pathways of kidney brain crosstalk during acute kidney injury (AKI). The release of cytokines, immune cells, uremic toxins and free oxygen and nitrogen radicals lead to inflammatory signals and disruption of BBB. Cytokines also activate neurotrophic factors and enhance the inflammatory signal pathways. The afferent-efferent signals mediate the signals of injury between kidneys and brain. (The arrows represent the induction of a process).

The kidney is innervated by a large number of sympathetic efferent nerves and peptidergic afferent nerves which play an important role in the neurological functioning of kidney. The nerves which travel from the peripheral organ (kidneys) toward the dorsal root ganglia along the spinal cord are called afferent sympathetic nerves while the efferent nerves carry neural impulses away from the central nervous system toward the peripheral organ (kidneys). There are few sympathetic nerves in the inner medulla of the renal vasculature, the highest innervation has been observed along the afferent glomerular arterioles followed by the efferent glomerular arterioles (85). Sympathetic nerve fibers are observed in cortex and pelvic regions of kidney whereas sensory fibers are seen in renal pelvic areas only (86) (Figure 3). The efferent nerves derived from para- and prevertebral ganglia, and enter the hilus of the kidney along the renal artery and vein, leading its fibers up to the renal arterioles, juxtaglomerular apparatus, and renal tubules (87). The sensory nerves enter parallel to the renal artery and ureter in the pelvis of the kidney and terminate as free nerve endings in the pelvic wall. They express α1 and β-adrenergic receptors which release catecholamines. The primary sensory neurons that innervate the kidneys are composed of peptidergic subpopulation of B-type primary afferents which give rise to unmyelinated and myelinated fibers (88). The sensory never fibers in the kidneys are mechanosensitive as well as chemo sensitive and contain substance P and CGRP (89).

In AKI, the sympatho-excitatory afferent nervous activity has been reported. Kidney disorders have been linked to excessive activity of renal sympathetic nerves and circulating catecholamines. It has been found that renal injury leads to increase in sympathetic nerve activity, by an increase in blood pressure, activation of immune system and modulation of inflammation (90). Sympathetic nerve activation (SNA) brings about a change in the renal blood flow and glomerular filtration rate through the innervated afferent/efferent arteriole (85). The increase in afferent renal nerve activity (ARNA) produced by the increased renal pelvic pressure leads to a reflex decrease in efferent renal sympathetic nerve activity (ERSNA), diuresis and natriuresis (91).

There is a feedback-based interaction between efferent renal sympathetic nerve activity (ERSNA) and afferent renal nerve activity (ARNA) called the reno-renal reflex, whereby an increase in the ERSNA stimulates an increase in ARNA. Furthermore, the increased ARNA exerts a negative feedback control of ERSNA to decrease its activity via activation of the reno-renal reflexes in the overall goal of upholding a low level of ERSNA (92). This feedback mechanism aids in the homeostasis by preventing overactivation of nerves and sodium retention. During kidney disease condition this feedback loop is weakened and the efferent nerve activity is not stopped leading to vasoconstriction, sympathoexcitation. This mediates RAAS activation and cerebral renin-angiotensin axis which promotes oxidative stress and injury (93). Renal nerves are also known to aid the migration of immune cells to kidney (94) and may trigger renal inflammation. Further, this inflammation leads to augmented ARNA leading to hypertension. Hence, renal denervation is considered a method to improve factors that include inflammatory cascade, reducing mesangial cell proliferation, lowering blood pressure and improving blood glucose levels relaying beneficial effects on renal functions.

Dense innervation of the juxtaglomerular cells and macula densa indicate that sympathetic nervous system (SNS) also plays a role in control of rennin release (95). On an increase in SNA, the urine volume decreases and Na^+^ reabsorption from proximal tubular cells increase (96). Along with this, the diameter of glomerulus decreases on activation of sympathetic nerves or on application of noradrenaline. An increase in adrenergic terminals in glomerulus could decrease the glomerular functions and thereby lead to loss of tubular and vascular functions (97). The podocytes, the cells of glomerulus that line the capillary loop, have the adrenergic as well as Angiotensin-II (Ang-II) receptors which causes constriction and lead to the development of glomerulosclerosis (98).

The renal afferent nerve relays information to the dorsal root ganglion and projects to neurons at central and peripheral nervous system (Figure 3). The activity of central sympathetic nervous system is increased in response to afferent nerve traffic. The presence of membrane-bound peptidases is thought to be the main mechanism determining the intensity and duration of the response to activation of primary afferent nerves by neuropeptides (99).

### Role of Neuropeptides in Pain Pathways During AKI

In kidneys, neuropeptides are secreted mainly from renal pelvic walls. This is an ideal location for mechanosensory nerves which sense stretch of the renal pelvic wall (89).

The mechanisms involved in the activation of the renal mechanosensory nerves takes place by stretching of the renal pelvic wall due to induction of cyclooxygenase-2 (COX-2) which leads to increased renal pelvic synthesis of PGE_2_. PGE_2_ increases the release of the neuropeptide “substance P” via activation of the cAMP-PKA signal transduction pathway. Substance P activates the afferent renal nerves by stimulating neurokinin-1 receptors in the renal pelvic area. However, it is reported that high AngII inhibits this afferent renal mechanosensory PGE_2_ release of Substance P via a pertussis toxin-sensitive mechanism (100).

Neuropeptides released by the nerves stimulate the pathways of inflammation, fibrogenesis of kidneys by distinct expression patterns within nociceptive neurons. Neuropeptide Substance P (SP) and CGRP (101) are known to play a role in AKI, both of which are known to be present in sensory nerves (but not in sympathetic efferent nerves). Some studies report that these neuropeptides stimulate the secretion of chemokines, cytokines and prostaglandin metabolites from immune cells or due to their interaction with immune cells or non-immune cells (102), while others show their protective role in AKI (73). On the contrary, it is postulated that NPY, somatostatin, α-MSH, ghrelin and Adrenomedullin (103) are associated with downregulation of inflammation in AKI.

The presence of substance P and CGRP containing afferent sensory nerves in the renal suggested a role for this neuropeptide in the activation of the afferent renal sensory nerves. The role of PGE2 has also come up in the activation of cAMP signal cascade in DRG neurons leading to depolarization of cell membrane and release of CGRP and Substance P, as well as stimulation of mechanosensory nerves by a calcium (Ca^++^)-dependent mechanism (104).

Among all other neuropeptides, CGRP is of importance in kidney diseases with regard to hemodynamic, potential role in immunity and pain sensation, paradoxically with both protective as well as non-protective roles in kidney injury (105). In general, CGRP is ubiquitously present in the body, generated from the alternative splicing of the calcitonin gene, both in the CNS and the PNS, but in kidneys CGRP is localized in capsaicin-sensitive nerve and in urogenital tract (106). CGRP causes increased blood flow and stable vasodilation in cerebral hemisphere, coronary, and kidney vascular beds (107). Due to its role in vasodilation, CGRP is the main neuropeptide responsible for inducing migraine headaches and neurogenic inflammation (108). CGRP immunoreactive nerves are associated with the cells such as merkel cells, melanocytes, mast cells, and keratinocytes, which are already stimulated during inflammation (109). The satellite glial cells also express receptors of CGRP. CGRP is co-stored and co-released with Substance P from capsaicin-sensitive peripheral afferent neurons in PNS. The trigeminal ganglia neurons release CGRP under conditions mimicking neurogenic inflammation (110). CGRP does not directly influence the basal blood pressure, but it is dependent upon factors such as angiotensin II, glucocorticoids, nerve growth factor, and sympathetic nerve reflexes. Its vasodilatation effects depend on other processes such as RAAS, oxidative stress and other inflammatory molecules. The feature that distinguishes CGRP from other vasodilators is its long duration of action (111).

CGRP is a key regulatory neuropeptide with important roles in immune outcome and inflammatory mediations such as with TNF-α (112). CGRP modulates an acute inflammatory response by controlling neutrophil recruitment. The immune cells have the receptor for CGRP. CGRP and TNF-α together demonstrate a bi-directional relationship between immune and nervous system (1, 113). There has been an inconclusive debate about CGRP's regulation by TNF-α. CGRP has been shown to exert either pro- or anti- inflammatory effects, depending on concentration, through the actions of TNF-α and/or IL-10. A study developed the claim that TNF-α causes an increase in CGRP promoter activity by activating the transcription factor NFkB, as well as the Jun N terminal kinase (JNK) and p38 mitogen-activated protein (MAP) kinase pathways (113). Along the similar lines, there is a claim that CGRP can downregulate TNF-α in macrophages, dendritic cells and other inflammatory responses. IL-6 which is markedly upregulated during pain and hyperalgesia also modulates the release of CGRP. Soluble IL-6 receptors called SIL-6R binds to IL-6 to form a complex SIL-6R/IL-6 which modulates the function of CGRP (114).

In AKI as well, the level of CGRP has a contradictory role. Most of the studies point toward the fact that CGRP is protective during AKI. The level of CGRP increases in the renal tissue that have undergone AKI and inhibits the TNF-α production by promoting endothelial PGI2 production (115). A contrary result in one of the studies claims that in spite of the high level of endogenous CGRP dosage, AKI could not be prevented. The possible explanations of non-protective role of CGRP are depletion of CGRP or the inhibition of CGRP production at the nerve ends (82). It is also possible that β_2_-adronoceptors on nerve cells inhibit CGRP release from sensory nerves (116).

The fact that renal denervation ameliorates AKI (117), and that androgenic receptors are affected during this disease, leads us to the inference that neurotransmitters and neuropeptides and their receptors might represent novel targets for AKI understanding. Hence these important messengers and pathways that work between the neural and immune response in context to AKI need to be explored.

CGRP is a downstream effector of TRPV1 channel on sensory neurons. TRPV1 is a molecular target of nervous system which is known for its role in immunity. Both CGRP and TRPV1 have been extensively studied in immune, pain sensitivity and visceral hypersensitivity (118). TRPV1 channel is activated by thermal stimuli (>43°C), protons, prostaglandins, lipids, low pH, chemical stimuli and inflammation (119). TRPV1 is also sensitized by Toll like receptors (TLR4) on macrophages (120) (Figure 2). The binding of these endogenous molecules activates the signaling cascade which releases inflammatory cytokines, resident immune cells, mast cells and neutrophils to stimulate release of CGRP (121). TRPV1 is overexpressed in DRG neurons and its signaling with CGRP is mediated by Erk, Akt, and CREB (Figure 2) (122). TRPV1 is postulated to have a therapeutic role in AKI (10, 82). The regulation of chemicals and protons on the receptors of TRPV1 is mainly done by alteration in the level of phosphorylation. TRPV1 monitors the regulatory pathways via the iCa^2+^ concentration through Phospholipase C (PLC) leading to decrease of phosphatidylinositol 4,5-bisphosphate (PIP2) level and through the production of diacylglycerol (DAG) (123). Inducible cyclic adenosine monophosphate early repressor (ICER) upregulates CGRP on binding to its receptors. This causes suppression of TNF-α during transcription aiding to some preventive effects during injury (10). In a reversal role, neuropeptide CGRP, SP, bradykinin, and inflammatory mediators sensitize TRPV1, activating the calcium flux leading to hyperalgesia and allodynia (124).

The immune cells share a bidirectional relationship with the nociceptor neurons. The nociceptors respond and interact with immune cells via inflammatory mediators. The release of cytokines, chemokines and nociceptors attract circulating cells to area of local inflammation and employ the cell bodies of nerves and DRG in neuroinflammation (125). The nociceptors like C-fibers release neuropeptides such as CGRP, Substance P or prostanoids. During injury, the peripheral nerve fibers and their cell bodies located in the dorsal root ganglion (DRG) and trigeminal ganglion signal the primary information about damage to the spinal and medullary dorsal horn. Satellite glial cells (SGCs) are connected to each other by gap junctions and it is postulated that during pain sensation, the gap between the satellite cells increases (126). The SGCs surround the DRG as well as the trigeminal ganglions and supply the nutrients to them. The SGCs also regulate the level of neurotransmitters and can affect the neuronal excitability through potassium buffering (127). The paracrine signaling is also one of the important ways of signal mediation in AKI (128). In trigeminal ganglions, the signaling between the neurons and SGCs contributes in nociceptor sensitization by involving PGE_2_ and neuropeptide CGRP. The CGRP released by the neurons affects the SGCs (101) and induces the production of IL-1β looping the process to inflammation and pain (129).

Kidney is a calcium-sensing organ that regulates the homeostasis of urinary and extracellular Ca^2+^ in body (130). Kidneys possess calcium sensing receptors (CaSR) across entire length of the nephron and thick ascending limb (131) and establish regulation of calciotropic hormones and renal feedback loop that incorporates the actions of the CaSR, parathyroid hormone (PTH) and calcitriol to provide rapid and local control of Ca^2+^ and Pi homeostasis (1, 132). AKI leads to rise in free intracellular calcium after AKI (Figure 2). This happens because of diminished ATP which reduces the calcium seizure within the endoplasmic reticulum as well as diminished release of cytosolic calcium into extracellular spaces. The process leads to instigation of proteases, phospholipases and cytoskeletal degradation. However, as a paradox, the increased intracellular calcium results in binding proteins such as Annexin which aid in recovery from AKI (133). Similar mechanism has been shown in previously on our models of polycystic kidney disease (PKD) where G protein signaling activates PLC, leading to release of Ca^2+^ from the internal stores by IP-3 dependent release. This is followed by entry of calcium through store activated Ca^2+^ channels, which stimulate calcineurin and NFAT (134).

Kidneys constitutively express endothelial NOs (eNOS) in endothelial cells and neuronal NOs (nNOS) in macula densa. During inflammation, inducible NO synthase (iNOS) is synthesized which leads to formation of Nitric Oxide (NO) and subsequent renal hemodynamic changes. NO acts as an antagonist of the vasoconstrictors rather than acting as a primary vasodilator and prevents glomerular thrombosis. However, the overproduction of NO during inflammation occurs via immunological NO synthase or neuronal NO synthase and leads to proximal tubular injury, neurodegenerative disease and apoptosis through the local generation of reactive nitrogen species (135).

### Changes Induced in Brain Due to AKI

One of the potential locations for the neuroimmune interactions during AKI is the brain itself. The neuropsychiatric disorders in response to kidney impairments is due to the vascular similarity of kidney and brain (136). It comprises of the neurons and the glial cells. The kidney glomeruli and brain share similar vascular supply and hence they are susceptible to similar microvascular pathologic processes. The glial cells are of three types: astrocytes, microglia and oligodendrocytes. All these central nervous system cells are surrounded by the tight and impermeable blood brain barrier. Under normal physiological conditions, the blood brain barrier (BBB) hampers the entry of circulating immune cells to access the brain regions making the immunological mechanism of brain different from peripheral tissues (137). The key players in the mediation of immune response are the microglia cells accounting for 5–20% of all cells in an adult human brain (138). The microglial cells are derived from distinct class of macrophage population and act like the first line of defense in the nervous system. In a normal condition, the microglial cells comprise of a small cell body and reticulated branches in multiple directions. These branches remain in fair motion to capture the signals relayed by the brain damage or the nervous system. As soon as the microglial cells receive signal, they take an ameboid form which enables these cells to ambulate freely through the brain. This response generated in response to the brain insult is known as microglial activation (139). Such microglial activation has been observed in brain in response to AKI causing morphological changes and secretion of cytokines and chemokines (140). An ischemic kidney injury model observed in rats shows that post-AKI, TLR4 levels increase in the hippocampus and striatum leading to AKI-induced encephalopathy (141). It was seen in nephrectomised rats that TNF and NF-κB were increased in the hippocampus and frontal cortex and this rise was associated with impaired memory and attention after 4 months (142). It has been demonstrated in another ischemia reperfusion injury (IRI) mouse model that AKI leads an increased gene expression of inflammation genes such as Cytotoxic T lymphocyte-associated protein 2 alpha, cell signaling genes like Map3k6 and extracellular matrix genes which consequently lead to hippocampal transcriptional dysregulation (143). Along with this, the matrix metalloproteinase-9 (MMP-9) whose expression increases during AKI, causes increased expression of CD11b and P38 mitogen activated protein kinases (p38MAPK) phosphorylation in microglial cells taking them to nociception during AKI (144).

Astrocytes are one of the most abundant cells which perform the homeostatic functions in the brain. They support the neurons by maintaining the BBB. The inflammation in astrocytes has been reported in AKI in animal model as well as in the older patients (145). Constitutive secretion of TNF-α has the ability to release glutamate from the astrocytes (146).

The oxidative stress developed due to AKI also leads to brain dysfunction. Reactive oxygen species (ROS) interacts with nitric oxide (NO) to generate toxic reactive nitrogen species, nitro tyrosine, in the cerebral cortex (147). The aggravation of inflammatory mediators, ROS and cytokines along with their decreased rate of clearance is associated with the BBB disruption. The increase in uric acid triggers a response which oozes out Weibel–Palade bodies by exocytosis (148). This mediates proinflammatory mediators including endothelin-1, IL-8 and angiopoietin-2 and bring changes in the BBB (149). The changes in vascular permeability of brain, protein leakage, alteration of essential amino acid concentrations, high serum sodium concentrations, inflammatory mediators and organic osmolyte are responsible for the BBB changes during (150). Increased formation of pro-inflammatory mediators concurring with their diminished clearance causes systemic inflammatory responses following AKI, associated with BBB disruption (151).

In brain, the movement of water across the plasma membrane is enabled by the aquaporin channels AQP4 and AQP9. During AKI, there is an increase in expression of aquaporin channels which facilitate influx of water into the brain by osmosis, leading to cerebral oedema (152). Experimental studies have reported that AKI causes bilateral renal ischemia (BRI) which impairs the learning, memorizing and motor activity due to increased level of BUN (153). Together this series of neuro-immuno-glial interactions amends the responses of somatosensory inputs leading to widespread, longer lasting allodynia, and hyperalgesia.

The association of brain and kidney has been determined during transplantation process. The brain death of the donor triggers the release of pro-inflammatory mediators, inflammatory molecules and adhesion molecules which affect the peripheral organs including kidney. A further research carried in rats show that before kidney transplantation, vagus nerve stimulation (VNS) of brain-dead donor rats improved the recipient and donor survival rates. A decrease in serum creatinine was found in rats undergone VNS as compared to the ones without having VNS (154).

Patients undergoing haemodialysis as a cure during renal injuries have been reported to develop brain injuries (155), cerebral ischemia (156), cerebral microbleeds (CMBs), and cognitive impairment such as dementia (157). Another study reports that, the dialysis receiving patients who had recovered from AKI developed more incidences of stroke and death as compared to patients without AKI (158). On the vice-versa role, the patients having TBI (acute cerebral damage) have been reported to have subclinical AKI. The patients have disrupted tubular cells, enhanced serum creatinine, increased urinary levels of low molecular weight proteins, apoptosis, pro-inflammatory phenotype and inflammatory chain reaction evidencing a crosstalk between kidney and brain (159).

## Discussion

A treasure of evidences and reports in research have demonstrated the multi-dimensional and entwined involvement of immune system and nervous system in generation and manifestation of pain. In longer-term implication, AKI leads to the shift of homeostatic balance, cognitive impairment, sleep disorders and chronic pain course-plotting the multi-organ damage. Pioneering studies which have been covered in this review, point to the fact that immune dysfunction instigate the neurological abnormalities during AKI and serve as a driving force for pain while the nervous system utilizes and responds via the cellular and molecular components of immune system for maintaining a homeostatic balance. These include interaction of microglia and astrocytes with cytokines and immune signaling molecules. The activated immune cells and impaired nerve activities along with modulation in neurotransmitters allow the immune cells to rupture and cross the blood brain barrier. Mechanistically, neuroinflammation causes manifestation of pain via central sensitization of sensory neurons, cytokines activity, chemokine proliferation and glial cells sensitization. Histological studies have revealed altered brain architecture and endothelial dysfunction during the pathogenesis of AKI. This neuro-immune interaction during AKI provides a pertinent example of complexities of inter-organ communication depicting that CNS is in constant dialog with peripheral immunity (Figure 3). The measurement of degree of inflammation and the role of antagonizing or targeting specific immune cells, cytokines or neuropeptides could lead to novel premises in treatment of AKI induced intensity and etiology of pain. More research into the inflammatory induced glial cells, nerve stimulated by immune cells or influence of endogenous neuropeptides that induce microglial activation seem to be a therapeutic approach in management of pain. Nevertheless, our understanding of the intricacies of the two system of neuro-immune induced pain is still limited and stands as a major challenge in the future. It generates an awareness that we should not focus on just the primarily diseased organ but consider holistic system-study approach in understanding the disease. Additionally, a universal interdisciplinary approach and understanding the robust targets must be exercised to achieve translational successes and drug development.

## Author Contributions

VP conceived the idea of AKI and pain pathways. AG worked as a research student on this idea to carry out important experiments to reach to develop this review article. SP developed the initial premise for setting up the model for the AKI along with researcher DK. All the drawings were developed and conceived together by VP and AG. All authors significantly read the article and contributed scientifically to bring it to this format.

## Conflict of Interest

The authors declare that the research was conducted in the absence of any commercial or financial relationships that could be construed as a potential conflict of interest.
